# Effect of felzartamab on the molecular phenotype of antibody-mediated rejection in kidney transplant biopsies

**DOI:** 10.1038/s41591-025-03653-3

**Published:** 2025-04-29

**Authors:** Matthias Diebold, Patrick T. Gauthier, Katharina A. Mayer, Martina Mackova, Christian Hinze, Jessica Chang, Uptal D. Patel, Ekkehard Schütz, Bernd Jilma, Eva Schrezenmeier, Klemens Budde, Georg A. Böhmig, Philip F. Halloran

**Affiliations:** 1 Division of Nephrology and Dialysis, Department of Medicine III, Medical University of Vienna, Vienna, Austria; 2 Clinic for Transplantation Immunology and Nephrology, University Hospital Basel, Basel, Switzerland; 3 Alberta Transplant Applied Genomics Centre, Faculty of Medicine and Dentistry, University of Alberta, Edmonton, Alberta Canada; 4 Department of Nephrology and Hypertension, Hannover Medical School, Hannover, Germany; 5 Human Immunology Biosciences, South San Francisco, CA USA; 6 Chronix Biomedical GmbH, Göttingen, Germany; 7 Department of Clinical Pharmacology, Medical University of Vienna, Vienna, Austria; 8 Department of Nephrology, Charité Universitätsmedizin Berlin, Berlin, Germany

**Keywords:** Medical research, Transcriptomics

## Abstract

A recent randomized controlled trial demonstrated that treatment with anti-CD38 monoclonal antibody felzartamab suppressed antibody-mediated rejection (ABMR) in kidney transplant patients but with recurrence after treatment in some patients. Here we examined the molecular effects of 6 months of felzartamab treatment on biopsies from the trial using genome-wide microarray analysis, comparing pretreatment, end-of-treatment (week 24) and posttreatment (week 52) biopsies from ten patients treated with felzartamab and ten patients in the placebo group. Felzartamab reduced molecular ABMR activity scores in all nine patients with baseline ABMR activity, selectively suppressing interferon gamma-inducible and natural killer cell transcripts, with minimal effect on ABMR stage-related endothelial transcripts. Suppression was often incomplete when ABMR activity was intense, and molecular recurrence was nearly universal by week 52. However, we also found that felzartamab had parenchymal benefits at week 52, slowing the trajectories of molecular injury scores beyond the treatment period, suggesting that suppression of ABMR activity could potentially slow future progression to kidney failure. These data provide preliminary molecular insights into the effects of CD38-directed treatment for ABMR, which have the potential to inform future therapeutic strategies.

## Abstract (web summary)

Molecular analyses of longitudinal kidney biopsies from a phase 2 trial testing an anti-CD38 antibody for kidney transplant rejection show temporary suppression of antibody-mediated rejection during treatment, with molecular recurrence at 52 weeks.

## Main

Antibody-mediated rejection (ABMR) represents a major cause of long-term kidney transplant loss^1,2^. ABMR manifests as microvascular inflammation (MVI) in peritubular capillaries (peritubular capillaritis, ptc) and glomeruli (glomerulitis, g), with slowly progressive glomerular and microvascular deterioration, nephron loss and kidney failure. ABMR is often associated with anti-human leukocyte antigen (HLA) donor-specific antibodies (DSA), and the pathogenesis is believed to involve natural killer (NK) cells engaging DSA bound to the microcirculation endothelium through their CD16a (Fc gamma receptor IIIA, FcγRIIIa) Fc receptors^3^. In the Molecular Microscope Diagnostic System (MMDx)^4–6^, ABMR activity manifests as increased ABMR activity transcripts: NK cell transcripts and interferon gamma (IFNγ)-inducible transcripts. ABMR also has time-dependent molecular stage changes, namely increased expression of ABMR-associated endothelium-expressed molecules. In MMDx, the molecular ABMR states are classified as early-stage ABMR (EABMR), with IFNγ-induced and NK cell expressed ABMR activity transcripts; fully developed ABMR (FABMR), which adds time-dependent ABMR-associated endothelial transcripts to activity changes; and late-stage ABMR (LABMR)^4^, with declining activity. The median times of diagnosis of these states in the INTERCOMEX study are approximately 1 year, 5 years and 8 years post-transplant, respectively, reflecting the temporal evolution of the ABMR process in the population, which may begin very early and silently and is often DSA negative^7^.

Until recently, no therapy had been proven effective in suppressing ABMR activity or halting progression^8,9^. However, a recent phase 2 trial of human IgG1 monoclonal anti-CD38 antibody felzartamab in kidney allograft recipients with active or chronic active ABMR has shown promise. The study addressed the safety, tolerability and efficacy of felzartamab therapy in 11 felzartamab-treated patients compared with 11 placebo-treated controls. Felzartamab treatment for 20 weeks suppressed histologic ABMR and decreased MVI substantially, with 7 out of 11 patients achieving an MVI score of zero at week 24, and a nonsignificant trend toward stabilization of estimated glomerular filtration rate (eGFR) at week 52. However, in the biopsy 32 weeks after the last treatment, there was histological recurrence in three patients. In addition, blood CD16^bright^ NK cells and donor-derived cell-free DNA, a blood-based test strongly associated with ABMR^10,11^, were suppressed by felzartamab, with partial recurrence after stopping treatment.

Molecular analysis of ABMR biopsies can provide insights into the mode of action of interventions targeting CD38 in ABMR^4^. While the previous study focused on clinical outcomes, the present study provides a comprehensive examination of the molecular features of the response in ten felzartamab-treated patients and ten placebo-treated patients from the trial with transcriptome analyses of biopsies for insights into the underlying mechanisms by which felzartamab mitigates ABMR, including its effects on injury repair responses, and documenting the extent of suppression and recurrence of molecular ABMR activity after treatment. All biopsies in the study were characterized in the genome-wide microarray-based MMDx system, which interprets measurements of gene expression using machine learning and unsupervised archetype classicification^4,12^. In addition to assessing ABMR activity, we assessed molecules that reflect recent parenchymal injury and correlate with risk of progression^13,14^. We also examined the possibility that felzartamab could trigger molecular T cell-mediated rejection (TCMR) activity.

## Results

**Fig. 1:**
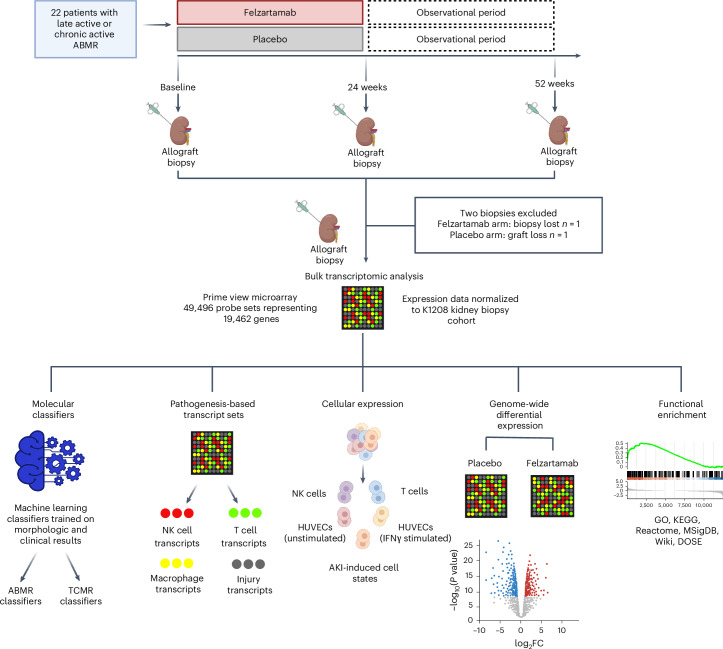
Study overview of the clinical trial. Created with BioRender.com.

The study participants were described in detail previously^15^. Comprehensive biobanking for all patients included three sets of blood and biopsy samples, including plasma donor-derived cell-free DNA as well as MMDx analysis of biopsies. For this analysis, 2 of the original 22 patients (one placebo, one felzartamab) had to be excluded, due to missing molecular biopsy results, leaving 10 felzartamab-treated and 10 placebo-treated patients (Fig. 1). Demographics of all 22 patients were published previously^15^ and are reproduced in Supplementary Table 1 for convenience. Baseline MMDx diagnoses for all ten felzartamab-treated patients were either FABMR (2, 3, 4, 7, 12 and 16), EABMR (13 and 22) or LABMR (9 and 19; Supplementary Table 2). However, patient 9 (LABMR) had minimal rejection activity despite being called active ABMR histologically. After treatment, MMDx found that all nine patients with molecular ABMR activity responded at week 24 and these same nine patients returned to a more active molecular class at week 52.

### Individual patient responses to treatment

The randomized controlled trial design of the felzartamab trial allowed us to monitor individual patient responses over time as well as calculate the formal treatment effect controlling for confounding effects of time (that is, time-related changes in the placebo group; Methods). Monitoring individual patient effects is critical for understanding the efficacy of the treatment given the underlying conditions for each patient, whereas the formal treatment effect provides an estimate of efficacy across the whole population. We defined the treatment effect for all variables as the interactive effect between treatment and follow-up and report it as delta delta (ΔΔ; that is, the difference between time-related changes among placebo and felzartamab groups; see equations (1)–(6) in Methods).

**Fig. 2:**
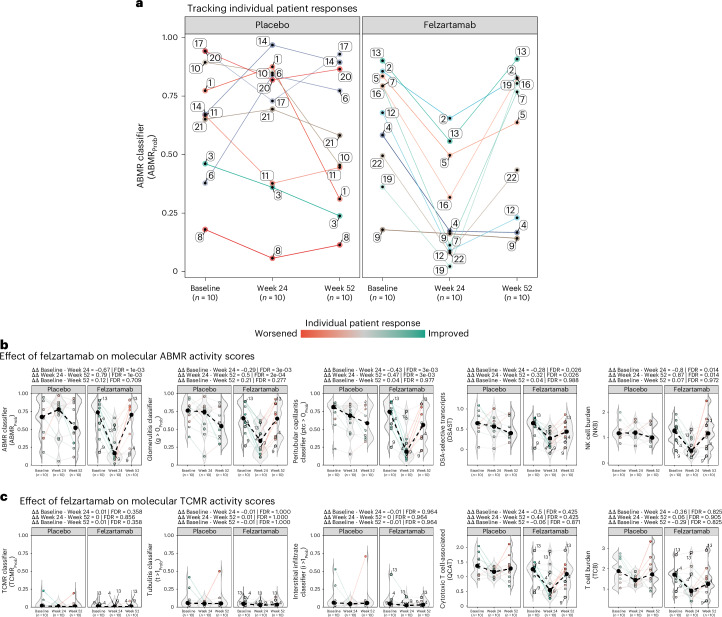
Effect of felzartamab on molecular ABMR and TCMR scores. **a**, Tracking individual patient scores for ABMR classifier (ABMR_Prob_). The solid circles represent individual patient scores for placebo (*n*_patients_ = 10) and felzartamab (*n*_patients_ = 10) arms across index, week 24 and week 52 biopsies. All biopsies represent biological replicates. The solid lines illustrate the change in score from baseline to week 24 to week 52 within treatment groups. **b**,**c**, The effect of felzartamab treatment on molecular ABMR (**b**) and TCMR (**c**) activity was assessed by ART-ANOVA. The effect of treatment was assessed as the interaction term between treatment group and follow-up visit. Two-tailed tests with *α* = 0.05 were used for all statistical testing, and *P* values were corrected for multiple testing using FDR. A detailed description of statistics for **b** and **c** is included in Extended Data Table 1. The shaded regions represent density estimates by group. The solid black circles represent the median scores by treatment group at baseline, week 24 and week 52. The dashed lines illustrate the changes in median scores (Δ) from baseline to week 24 to week 52 within treatment groups. The solid colored circles represent individual patient scores. The numbers identify patients of interest. The solid colored lines illustrate the change in individual patient scores from baseline to week 24 to week 52.

At baseline, 9/10 biopsies in the felzartamab group had molecularly active ABMR_Prob_ scores, the exception being patient 9 as mentioned above^15^ (Fig. 2a; for a definition of molecular scores, see Supplementary Table 3). ABMR_Prob_ scores in all nine felzartamab-treated patients with elevated scores were suppressed at week 24. Responses were incomplete in some felzartamab-treated patients with high baseline scores (that is, patients 2, 5 and 13). Eight of the responders (all but patient 4) exhited an increase in the molecular ABMR_Prob_ score by week 52. Patient 9, with low baseline activity, did not show an increase in ABMR activity after treatment.

### Felzartamab suppresses all molecular ABMR activity scores

We assessed the therapeutic effect of 20 weeks of felzartamab treatment on five ABMR scores for baseline, week 24 and week 52 biopsies: three classifiers (ABMR_Prob_, g >0_Prob_, ptc >0_Prob_) and two pathogenesis-based transcript sets (PBTs); DSA-selective transcripts and NK cell burden (NKB). All were elevated at baseline in all felzartamab-treated patients at baseline, the exception again being patient 9. In all nine patients with elevated baseline ABMR activity, ABMR activity scores were suppressed by felzartamab treatment at week 24 (Fig. 2b and Extended Data Table 1). After cessation of felzartamab therapy at week 20, there was a relapse in ABMR activity scores by week 52 in eight of the nine felzartamab responders, the exception again being patient 4.

Before the felzartamab study trial, there was concern about the potential of felzartamab therapy to trigger TCMR^16–18^. Accordingly, we assessed the therapeutic effect of felzartamab on five molecular scores reflecting TCMR activity (Fig. 2c and Extended Data Table 1). There was no significant effect, neither on classifiers (TCMR_Prob_, tubulitis (t) lesion score > 1 (t >1_Prob_), interstitial inflammation (i) score > 1 (i >1_Prob_)) nor on TCMR-related transcript sets (that is, T cell burden and cytotoxic T cell-associated transcripts). Note the high T cell burden and cytotoxic T cell-associated transcripts in patient 13, suggesting that this patient had subthreshold TCMR-like activity at baseline. The occurrence of subthreshold T cell transcripts in patient 13 prevents the interpretation of NK cell transcripts (NKB) owing to the sharing of transcripts between T cells and NK cells.

**Fig. 3:**
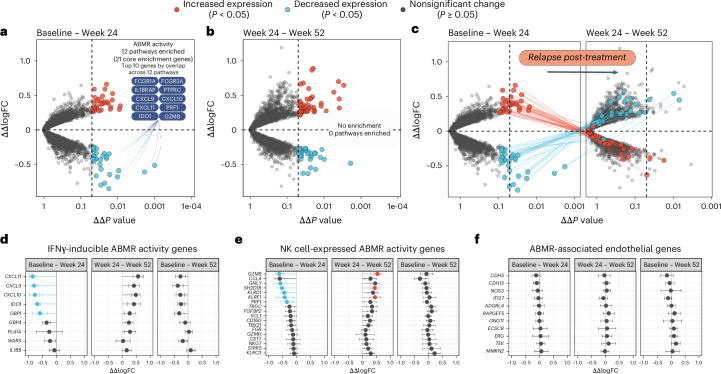
Genome-wide effect of felzartamab treatment on kidney allograft biopsy gene expression. **a**–**c**, The effect of felzartamab treatment on gene expression (ΔΔlogFC) from baseline to week 24 (**a**), week 24 to week 52 (**b**) and baseline to week 52 (**c**). The circles represent the ΔΔlogFC (*y*-axis) and *P* values (x-axis) of individual genes compared between biopsies from placebo (*n*_patients_ = 10) and felzartamab (*n*_patients_ = 10) arms from baseline to week 24, and week 24 to week 52. All biopsies represent biological replicates. Functional enrichment by differentially expressed genes is included in **a**–**c**. The gene labels represent the top ten genes by representation across all enrichment terms. **d**–**f**, The effect of felzartamab on IFNγ-inducible ABMR activity genes (**d**), NK cell-expressed ABMR activity genes (**e**) and ABMR-associated endothelial genes (**f**). Data in **d**–**f** are represented as fold change ± empirical Bayes moderate standard errors in fold change calculated using moderated Bayes *t*-tests.

We then carried out a genome-wide transcriptome analysis to assess the effects of felzartamab on the expression of individual genes, again specifying a formal treatment effect to account for time-dependent changes in the placebo group. Genes were assessed by their rank order of uncorrected *P* values. Ten of the top 20 ranked genes between baseline and week 24 were decreased (Fig. 3a, blue symbols) and were previously annotated as correlating with ABMR activity in the INTERCOMEX study^19^ and either inducible by IFNγ (for example, *CXCL10*, *CXCL11*, *GBP1* and *IDO1*) or highly expressed in NK cells (for example, *SH2D1B*, *FCGR3A* and *GZMB*; Extended Data Table 2).

These findings were bolstered by functional enrichment analysis of Gene Ontology (GO), Reactome, Kyoto Encyclopedia of Genes and Genomes (KEGG), Molecular Signatures Database (MsigDB), Disease Ontology Semantic and Enrichment analysis (DOSE) and WikiPathways libraries. Between baseline and week 24, 12 pathways related to ABMR activity were suppressed (Extended Data Table 3), reflecting the suppression of NK- and IFNγ-induced genes. Functional enrichment did not detect any previously annotated processes associated with the genes with increased expression at week 24 (Fig. 3a, red symbols), compatible with these increases reflecting the ability of felzartamab to reverse the suppression of these genes by ABMR activity. Among the top 20 ranked genes between baseline and week 24, 10 were increased and may reflect recovery of expression of normal parenchymal genes that had been suppressed by ABMR activity. All decreased in expression as ABMR activity returned by week 52 (Fig. 3b, red symbols).

Between week 24 and week 52, functional enrichment analysis did not detect any enriched terms. However, when comparing the top-ranked genes between week 24 and week 52 to the immune-response gene expression profile observed between baseline and week 24, most genes that were decreased (blue) or increased (red) between baseline and week 24 showed reversed changes from week 24 to week 52 (Fig. 3c).

### Felzartamab suppressed IFNγ-inducible and NK cell genes

Felzartamab suppressed the expression of IFNγ-inducible ABMR activity genes (Fig. 3d and Extended Data Table 4) and NK expressed ABMR activity genes (Fig. 3e and Extended Data Table 5) but had little effect on ABMR-associated endothelial genes (Fig. 3f and Extended Data Table 6). The therapeutic effect of felzartamab suppressed IFNγ-inducible ABMR activity genes by 47% compared with baseline, but by week 52 they increased back by 30% compared with week 24. Although still 17% below baseline at week 52, the difference was not statistically significant. The therapeutic effect of felzartamab also suppressed NK expressed ABMR activity genes by 28% compared with baseline, followed by an increase of 27% by week 52 compared with week 24. There was no effect of felzartamab therapy on ABMR-associated endothelial genes across any time window (Extended Data Table 6).

We examined the top 20 genes that were increased at week 24 after felzartamab therapy (Extended Data Table 7). Note that *TRAV12-3* has very low expression and may be unreliable. All others either correlated negatively with ABMR activity genes or correlated positively with ABMR-associated endothelial genes, which show reduced expression in human umbilical vein endothelial cells (HUVECs) after IFNγ treatment, or both. These findings suggest that the increased genes (Fig. 3a, red symbols) reflect the removal of inhibition by ABMR activity due to felzartamab effects.

### Felzartamab treatment reduces long-term parenchymal injury

**Fig. 4:**
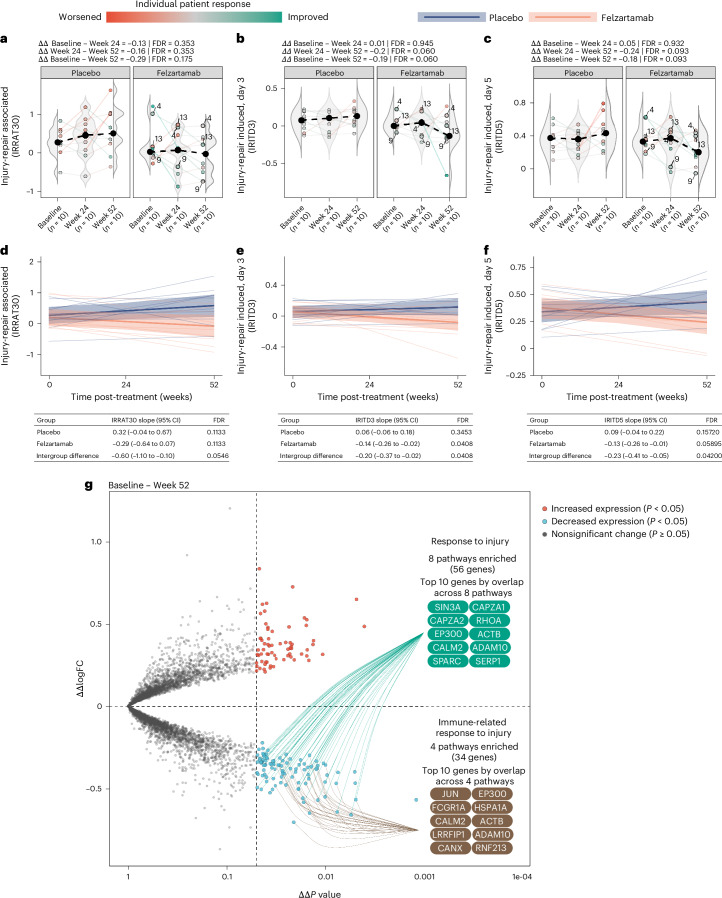
Effect of felzartamab treatment on molecular injury scores. **a**–**c**, The effect of felzartamab on IRRAT30 (**a**), IRITD3 (**b**) and IRITD5 (**c**) molecular injury scores. The shaded regions represent density estimates by group. The large circles represent the median score for placebo (*n*_patients_ = 10) and felzartamab (*n*_patients_ = 10) arms across index, week 24 and week 52 biopsies. All biopsies represent biological replicates. The dashed lines illustrate the change in median score (Δ) from baseline to week 24 to week 52 within treatment groups. The colored circles represent individual patient scores. The solid lines illustrate the change in individual patient scores from baseline to week 24 to week 52. The effect of felzartamab treatment on molecular injury scores was assessed by ART-ANOVA. The effect of treatment was assessed as the interaction term between treatment group and follow-up visit. Two-tailed tests with *α* = 0.05 were used for all statistical testing, and *P* values were corrected for multiple testing using FDR. **d**–**f**, Mixed effect regression modelling of the effect of felzartamab on IRRAT30 (**d**), IRITD3 (**e**) and IRITD5 (**f**) molecular injury scores. The thin solid lines represent individual patient trajectories for placebo (black) and felzartamab-treated patients (red). The thin dotted line depicts patient 9. The thick solid lines represent the predicted score by linear mixed-effects model fit with an interaction term between the score and time, including time and patient as random effects (FDR_IRRAT30_ 0.0546, FDR_IRITD3_ 0.0408, FDR_IRITD5_ 0.0420). The shaded regions depict 95% confidence intervals for predicted scores. **g**, Genome-wide effect of felzartamab treatment on gene expression (ΔΔlogFC) from baseline to week 52. Functional enrichment by differentially expressed genes is shown from baseline to week 52. The gene labels represent the top ten genes by representation across all enrichment terms. CI, confidence interval.

We studied the therapeutic effects of felzartamab on injury-induced gene sets, which are of particular interest because they strongly predict survival in kidney transplants with ABMR^13^. IRRAT30, IRITD3 and IRITD5 scores in felzartamab-treated patients were lower at week 52 versus week 24, and week 52 versus baseline (although not significant), suggesting the possibility of some resolution of injury after felzartamab treatment (Fig. 4a–c).

Given the apparent linear relationship of injury scores over time (that is, compared with the unimodal response seen in ABMR scores), we examined the slope of the injury scores in felzartamab biopsies versus placebo biopsies with a linear mixed-effects model. The slopes of injury-repair response-associated transcripts (IRRAT30), injury and repair induced transcripts, day 3 (IRITD3), and injury and repair induced transcripts, day 5 (IRITD5) scores in patients treated with felzartamab were negative in patients treated with felzartamab and positive in patients treated with placebo (Fig. 4d–f).

We then assessed the genome-wide effects of felzartamab over the entire study period (that is, baseline to week 52). Among the top 20 ranked genes (Fig. 4g and Extended Data Table 8), 17 were previously annotated as injury-inducible genes and were decreased at week 52, suggesting resolution of injury from the parenchymal effects of ABMR as a durable late consequence of 24 weeks of felzartamab therapy.

Genes *AADACL4* and *RERGL*, both suppressed by acute kidney injury (AKI) and ABMR, were increased at week 52, also suggesting recovery from injury. Moreover, the top 20 ranked injury-related genes annotated by Hinze et al.^20^ all showed decreased expression at week 52 compared with baseline (ΔΔfold-change (FC); Extended Data Table 9). Seven had been assigned to AKI-associated cell states previously linked to fibrosis, epithelial–mesenchymal transition and failed repair^20–22^. These findings were further supported by functional enrichment analyses, in which 12 terms depicting a response to injury were suppressed 32 weeks after treatment (Extended Data Table 10). Of note is the strong effect of treatment on the pathway represented by injury molecules identified by Hinze et al. in single-nucleus studies representing the common epithelial response to injury in human kidneys with AKI^20,23^.

Taken together, the molecular evidence indicates that treatment by felzartamab not only suppresses ABMR activity genes at week 24, but also leads to sustained resolution of expression of injury-induced genes at week 52, 32 weeks after therapy was stopped.

## Discussion

We applied MMDx to analyze the transcriptome in biopsies of patients receiving felzartamab anti-CD38 antibody for the treatment of histologically defined active or chronic active ABMR, including ten felzartamab-treated patients and ten placebo-treated patients from a randomized controlled trial^15^. The main effect was a consistent decrease in ABMR activity-related classifiers and gene set scores in patients treated with felzartamab, with no effect on the scores for TCMR. Felzartamab suppressed ABMR activity scores in all nine patients who had elevated baseline molecular ABMR activity (except patient 9 who had histologic but minimal molecular activity^15^). Felzartamab suppressed the expression of IFNγ-inducible and NK-expressed ABMR activity genes with little effect on ABMR-associated endothelial genes such as *ROBO4*. As seen molecularly, felzartamab response was universal (9/9) in patients with molecularly active ABMR. Responses were often incomplete when baseline activity was very high, which is evident in the median treatment effects (that is, ΔΔ scores; most evident in felzartamab-treated patients 2, 5, 13 and 16). After cessation of treatment, relapse was near universal (8/9 responders who had molecular ABMR activity at baseline). Of interest, patient 4 who did not relapse also did not show recovery of blood CD16^bright^ NK cells^15^, whereas all other patients had some recovery of blood NK cells at week 52. ABMR activity scores returned to levels similar to those in the baseline biopsies of 8/9 responders. We also found that felzartamab compared with placebo ameliorates parenchymal injury-induced changes, which may be related to the observed trend toward stabilizing eGFR^15^. The results of this comprehensive analysis of gene expression patterns in the context of an interventional trial with systematic tissue collection reinforces the potential of felzartamab as a therapeutic option for patients with ABMR and suggests the additional benefit of recovery of ABMR-induced injury.

These results show how genome-wide MMDx analysis of biopsies can add insights to trial results. The treatment response and relapse were consistent within classifiers and gene sets reflecting ABMR activity, generally agreeing with the previously reported histologic activity measures (g/ptc/MVI scores)^15^, with the major difference being that molecular assessment detected more responses, indicated some incomplete responses and showed a higher incidence of relapse at week 52. This suggests that molecular testing is more sensitive and more able to quantify responses and recurrences. There was a lack of effect of felzartamab on the expression of ABMR-associated stage-related endothelial genes, like the histological endothelial lesions such as glomerular double contours and ptc-basement membrane multilayering, probably because the endothelial ABMR-induced transcripts reflect stage rather than activity and will resolve slowly if they resolve at all. Longer-term observations will be needed to determine whether the molecular and histologic endothelial changes of ABMR (such as glomerular double contours) will heal if ABMR is permanently suppressed. Gene expression profiling (for example, MMDx) will be particularly useful in this regard, as in previously published analyses^24^.

The decline in biopsy NK cell transcripts after felzartamab treatment, as well as the previously reported reduction of peripheral blood CD16^bright^ NK cell counts^15^, suggests that the main mechanism of action of felzartamab is on NK cells and that this effect recovers slowly and incompletely (for example, patient 4). The effect of felzartamab could be to kill NK cells, to change their trafficking or to inactivate them^15^, and return of ABMR activity is probably dependent on the recovery of blood NK cells. The top transcripts suppressed after 20 weeks of felzartamab treatment were highly expressed in NK cells or were inducible by the IFNγ (and perhaps other cytokines) released from NK cells. These results align with the hypothesis that the main therapeutic effect of felzartamab in ABMR is suppression of NK cells rather than killing plasma cells and reducing DSA, but does not exclude additional effects on plasma cells, potentially through anti-CD38-mediated killing of the plasma cells by NK cells. The mode of action of felzartamab may be different from that of other anti-CD38 antibodies such as daratumumab and isatuximab, which are also being studied in the context of ABMR treatment and desensitization^25,26^. Unlike those antibodies, felzartamab cannot mediate complement-dependent cytotoxicity, instead inducing its effects through antibody-dependent cellular cytotoxicity and phagocytosis. It is speculated that the elimination of NK cells by anti-CD38 monoclonals may impair antibody-dependent cellular cytotoxicity and, in the absence of complement activation, reduce effects on CD38-positive plasma cells^15^.

It is encouraging that we found no evidence that felzartamab activates undesirable T cell responses, even in patient 19 who had some baseline elevation of T cell transcript expression. Case reports and evidence from animal models have suggested that treatment with anti-CD38 could trigger TCMR through the depletion of regulatory T and B cells^16–18^. However, the present detailed analysis of the therapeutic effect in individual patients, like the prior publication^15^, found no meaningful changes in TCMR activity.

These findings underscore the potential for felzartamab to serve as a probe to discover new aspects of the molecular biology of ABMR. The increased expression of some genes at week 24 indicates that ABMR activity at baseline was suppressing the expression of these genes and that inhibiting ABMR activity can restore the expression of these genes, indicating that ABMR activity had more widespread effects than had previously been appreciated. We had already noted that the ABMR-associated endothelial genes were generally reduced in expression by IFNγ in HUVECs in vitro. In our largest reference set of 5,086 kidney allograft biopsies^19^, many of the increased genes at week 24 correlated with the ABMR-associated endothelial genes or correlated negatively with ABMR activity genes. Expression of these genes increased at week 24 after felzartamab therapy but then decreased as the ABMR activity relapsed by week 52.

The effect of felzartamab therapy at week 52 included a reduction of injury scores that correlate with prognosis in ABMR^13^, offering hope that felzartamab treatment will not only reduce ABMR activity but also reduce late kidney transplant failure by permitting slow resolution of ABMR-induced injury and arresting new injury. A number of injury-induced genesets and pathways had reduced expression after felzartamab at week 52, including some annotated in common epithelial response patterns to human acute kidney injury^20^, suggesting a unexpected benefit of felzartamab therapy. This effect was delayed compared with the therapeutic effect on ABMR activity, and a longitudinal effect of felzartamab therapy was detected when observing the slopes of the molecular injury scores over the course of the 52-week study period. The slope of the injury scores over 12 months was negative in patients who received felzartamab, whereas it was positive in patients in the placebo arm, suggesting that the course of felzartamab therapy reduced the risk of progression.

Genome-wide differential expression and enrichment analyses provided a similar interpretation regarding the therapeutic effect of felzartamab. Between baseline and week 24, multiple pathways related to ABMR activity were suppressed, and some parenchymal genes showed increased expression, presumably because they had been suppressed by the ABMR activity. However, from baseline to week 52, the core enrichment genes from those pathways had almost no overlap with the core enrichment genes involved in the suppression of immune-related pathways seen from baseline to week 24. This suggests that the resolution of injury-induced changes (positive, resolution of normal functions; negative, resolution of injury-induced changes) occurs over a longer time. Thus, a 24-week course of felzartamab to suspend ABMR activity, even if temporary, may have an enduring benefit even as the ABMR activity slowly reemerges.

This phase 2 clinical trial has a number of limitations. The study population was small, and validation of these findings in ensuing larger clinical trials for felzartamab will be anticipated with interest to confirm not only the suppression of ABMR activity but also the resolution of ABMR-induced parenchymal injury^13,14^. Small sample sizes reduce the power to detect relevant differences and potentially add to false discovery. Nonetheless, despite the small sample size and adjusting the hypothesis testing for multiplicity, we detected substantive statistically significant effect sizes for molecular scores. Genome-wide assessment in this small cohort was a challenge because differential expression for individual genes did not pass FDR correction. As a result, we did not focus our interpretation on treatment effects for individual genes and instead focused on rank ordering by *P* value as well as bulk trends revealed by gene set enrichment analysis based on rank-ordered genes as opposed to genes selected by cutoff. The consistency in the overarching interpretation of the molecular findings was reassuring, particularly in light of the agreement between molecular findings with clinical findings at week 24. Although we found that felzartamab suppressed the NKB scores and expression of NK cell-expressed antibody-mediated rejection activity genes, we could not directly interrogate the effects of felzartamab on CD16^bright^ NK cells present in the biopsy, and future single-cell studies addressing the effect of felzartamab on NK cells would be of interest, particularly with respect to the time course of relapse after treatment. The exclusion of 2 of the original 22 patients because of missing molecular biopsy results (1 patient in the placebo group who experienced graft loss before the scheduled allograft biopsy at 24 weeks and 1 patient in the treatment group because the biopsy was lost in shipment) are unlikely to have introduced selection bias. Lastly, the microarray platform used here for molecular measurements has limitations, and detailed RNA sequencing and single-cell sequencing in the future will undoubtedly add valuable insights.

While the present analysis adds to the previously published results in providing hope that felzartamab will change ABMR outcomes^2^, we are eager to resolve many key issues. How long must treatment be continued or how frequently must treatment be repeated? Will patients with incomplete responses be stabilized or will they progress despite treatment? If responses are incomplete, what else can be added? Does the effect of felzartamab on plasma cells and the mild reduction in DSA contribute to the therapeutic effect^15^? Felzartamab was effective in all patients who had molecular ABMR activity but did not induce substantial long-term modification of the ABMR disease state after treatment was discontinued, suggesting an opportunity for additional interventions, including ongoing felzartamab dosing. The suppression of individual injury genes and pathways at week 52 raises the possibility that 20 weeks of felzartamab treatment may have beneficial effects on the parenchyma that endures up to 32 weeks after treatment, but the implication is that felzartamab use may have to be repeated or continued to change outcomes, and the next generation of trials must be designed to answer these questions. It will also be of interest that felzartamab-treated patient 4 did not develop recurrent ABMR activity, suggesting that relapse may not be universal, perhaps dependent on blood NK cell recovery.

## Methods

### Ethics approval

Ethics approval was obtained from the institutional ethics committees of the Medical University of Vienna and Vienna General Hospital (EK1161/2021) and Charité Universitätsmedizin Berlin (EUDRACT identifier 2021-000545-40, code FELZ01). Informed consent was obtained from all patients before enrollment as approved by the local center institutional review board, and patients were not compensated for their participation in the study. The trial protocol was approved by local ethics committees at all trial sites and by federal regulatory agencies^15^. The trial adhered to the principles of Good Clinical Practice, Good Laboratory Practice, the Declaration of Helsinki and the Declaration of Istanbul. Full details regarding the trial, including ethics compliance and committees, can be found at EUDRACT (EudraCT number 2021-000545-40) and ClinicalTrials.gov (NCT05021484).

### Trial design

This analysis was a prespecified endpoint of an investigator-driven, randomized, double-blind, placebo-controlled phase 2 trial, conducted to evaluate the safety, tolerability and efficacy of felzartamab in kidney allograft recipients diagnosed with ABMR (EudraCT number 2021-000545-40, NCT05021484)^27^. Details of the trial design and main trial results have been reported previously^15,27^. Twenty-two patients with late active or chronic-active ABMR were randomized to receive felzartamab or placebo (Fig. 1). Treatment was administered for 20 weeks, followed by a 6-month observation period. Patients were scheduled to undergo systematic follow-up biopsies at baseline and 24 and 52 weeks after trial initiation.

Patients were eligible if they had a functioning kidney allograft ≥180 days after transplantation with an eGFR ≥20 ml min^−1^ 1.73 m^−2^. ABMR diagnosis was based on the Banff 2019 schema^28^ and presence of HLA class I and/or II antigen-specific antibodies (preformed and/or de novo). Major exclusion criteria included the presence of TCMR Banff grade ≥1, de novo or recurrent severe thrombotic microangiopathy, polyoma virus nephropathy or de novo or recurrent glomerulonephritis. Detailed eligibility criteria are published elsewhere^15^. For the present study, which focused on the molecular results of study biopsies, we excluded two patients because of missing biopsy results. One patient in the placebo group lost his graft after 14 weeks owing to ABMR, and no 24- and 52-week follow-up biopsies were performed; for one patient in the felzartamab group, no molecular analysis at week 24 was available.

As described in detail elsewhere, patients received a total of nine doses of intravenous felzartamab (16 mg kg^−1^ per dose) or matching placebo over the course of 20 weeks. The first four doses were given weekly, followed by monthly doses. Premedication with diphenhydramine, paracetamol and prednisolone or matching 0.9% saline was given 30 min before the first two felzartamab or placebo infusions. Study outcomes have been detailed previously^15^. For these analyses, we focused on the evolution of gene expression patterns under treatment with felzartamab as well as their association with graft.

The primary outcome of the trial was the safety and tolerability of felzartamab in kidney transplant recipients with ABMR on baseline immunosuppression. Secondary outcomes included assessments of DSA and immunoglobulin levels (at weeks 0, 12, 24 and 52), immunodominant DSA (mean fluorescence intensity, dilution-based changes and number detected) and total Ig/IgG subclasses. Additional outcomes involved evaluating leukocyte subsets, circulating immune cells and CD38 expression using flow cytometry, as well as results from protocol biopsies (at weeks 24 and 52), focusing on ABMR classification, inflammation scores (g+ptc, chronic glomerulopathy, interstitial fibrosis) and molecular analyses (MMDx and rejection-related classifiers). Biomarkers such as *CXCL9*, *CXCL10*, *BAFF* and *TTV* were measured alongside clinical parameters, including eGFR slope, iBox scores, proteinuria and 12-month graft or patient survival. Further information is provided by Mayers et al.^27^ (including supplementary appendix and statistical analysis plan). Patient age and sex was collected and reported in the study, but no age- or sex-based analyses were carried out in the study, in part because of insufficient sample sizes (Supplementary Table 1).

### Biopsies

The present analysis included 60 biopsies performed in 20 patients (3 biopsies per patient, at baseline, 24 weeks and 52 weeks). All biopsies were analyzed following the 2019 update of the Banff classification^28^. For each biopsy, a 3-mm portion of one core was placed immediately in RNAlater and shipped at ambient temperature to the Alberta Transplant Applied Genomics Centre (University of Alberta, Edmonton, Alberta, Canada) for microarray analysis by MMDx. The workflow and technology have been described previously^29–32^. In brief, gene expression was measured using GeneChip, PrimeView U219 arrays (Applied Biosystems, Thermo Fisher Scientific). All gene expression data were collected using Affymetrix GeneChip Command Console Scan Control v.4.0.0.1567 and processed in R using the BioBase v.2.64.0 library. Gene expression data were normalized to the K1208 reference set of kidney allograft biopsies using robust multiarray averaging as a required preprocessing step for assessment by MMDx (this includes prediction of classifier scores trained on K1208 data)^4^. Biopsies were then assessed with a suite of machine-learning-derived classifier scores and PBT scores to guide an expert reader at making a molecular diagnosis.

### Definition of classifiers, PBTs and selective gene sets

All classifier and PBTs were previously developed using kidney transplant biopsies or murine models (Supplementary Table 3). Four new gene sets were defined to depict ABMR-activity (Supplementary Table 4), ABMR-activity genes, IFNγ-inducible ABMR activity genes (Supplementary Table 5), NK cell-expressed ABMR activity genes (Supplementary Table 6) and ABMR-associated genes expressed in endothelial cells (Supplementary Table 7). ABMR-activity genes were selected as the top 20 genes correlated with early ABMR (EABMR) score in a kidney cohort of 5,086 biopsies (K5086)^19^. IFNγ-inducible ABMR activity genes were selected by taking the top 100 genes correlated with EABMR score with a Spearman correlation >0.2 in K5086, retained genes with expression in HUVECs fivefold greater than their expression in NK cells and IFNγ-stimulated HUVECs in the study population^3^. NK cell-expressed ABMR activity genes were isolated by taking the top 100 genes correlated with EABMR score in K5086 and removing genes with unstimulated and IFNγ-stimulated HUVEC expression >50th percentile in the study population, then retaining the top 20 genes by expression in NK cells. ABMR-associated endothelial genes were selected by taking the top 100 genes correlated with FABMR score K5086, then retaining genes that had fourfold greater expression in unstimulated HUVECs than NK cells.

A final gene set depicting genes involved in acute kidney injury was prepared by isolating marker genes from AKI-induced cell states in kidney allografts^20^. We collected all marker genes reported in ‘Additional file 7: Extended Data Table 2’ from Hinze et al.^20^, and screened the reported cell types to retain only ‘New’ cell states (that is, those induced by acute kidney injury). The cell state annotations were included for interpretation of individual genes. For functional enrichment analyses, all of the marker genes were collated to produce a single gene set.

### Statistical analyses

Continuous variables are summarized as medians and interquartile range and discrete variables as counts and/or percentages. A two-tailed approach with *α* = 0.05 was used for all statistical testing, and *P* values were corrected for multiple testing using false discovery rate (FDR). Statistical analyses were performed using R software, v.R 4.3.3.

#### Omnibus test on molecular score categories

To detect if there were global effects across groups of molecular scores by phenotype annotation, permutational multivariate analysis of variance was carried out for each annotation grouping (that is, all ABMR, TCMR, macrophage, atrophy-fibrosis and parenchymal scores) using the vegan v.2.6.8 package in R^33^ using Euclidean distances and 10^6^ permutations.

#### Treatment effect on molecular classifier and PBT scores

The effect of felzartamab treatment on classifier and PBT scores was assessed by an aligned-rank transformed analysis of variance (ART-ANOVA)^34,35^. The effect of treatment was assessed as the interaction term between treatment group and follow-up visit. The random effect of patient was included in the model to handle repeated measurements (equation (1))

1 $$\begin{array}{l}{Y}_{ijk}=\mu +{\mathrm{Treatment}}_{i}+{\mathrm{Followup}}_{j}\\\qquad+\,{\text{Treatment:Followup}}_{ij}+\left(1\,|\,{\mathrm{Patient}}_{k}\right)+{\varepsilon }_{ijk},\end{array}$$

after which all pairwise contrasts within the interaction term (that is, Treatment:Followup_*ij*_) were carried out to define the effect of treatment^36^. To calculate the trajectories of injury scores, a linear mixed-effects model was fit with an interaction term between the score and time^37^, including time and patient as random effects (equation (2)) using the lmerTest v.3.1.3 and ggeffects v.1.7.1 packages in R. The assumptions of the linear mixed-effects model were confirmed using diagnostic plots and statistical tests provided by the ‘performance’ package 0.12.3 in R.

2 $$\begin{array}{l}{Y}_{ijk}=\mu +{\mathrm{Treatment}}_{i}+{\mathrm{Followup}}_{j}+\text{Treatment:Followup}_{ij}\\\qquad+\,\left({\mathrm{Followup}}_{j}\,|\,{\mathrm{Patient}}_{k}\right)+{\varepsilon }_{ijk}.\end{array}$$

#### Treatment effect on gene expression

We also assessed the effect of felzartamab treatment on genome-wide gene expression using 4,838 interquartile-range-filtered genes. Genes that differed at baseline between placebo and felzartamab arms (*P* < 0.05) were excluded from interpretation. Differential expression among treatment arms for each follow-up visit was calculated using the limma v.3.60.4 package in R^38,39^, with ‘%cortex’ included as a covariate in the model to partly correct for the high composition of medulla in some biopsies when determining differential expression (equation (3))

3 $$\begin{array}{l}{Y}_{ijk}=\mu +{\mathrm{Treatment}}_{i}+{\mathrm{Followup}}_{j}+\text{Treatment:Followup}_{ij}\\\qquad+\,\%{\mathrm{cortex}}_{k}+{\varepsilon }_{ijk}.\end{array}$$

Specific contrasts were then defined to isolate the interactive effect of treatment (ΔΔFC) and tested using the empirical Bayes method (equations (4)–(6))

4 $$\begin{array}{l}\Delta \Delta {\mathrm{FC}}_{{\mathrm{baseline-week24}}}=\left({\mathrm{Felzartamab}}_{{\mathrm{week24}}}-{\mathrm{Felzartamab}}_{{\mathrm{baseline}}}\right)/2\\\qquad\qquad\qquad\qquad\quad-\,\left({\mathrm{Placebo}}_{{\mathrm{week24}}}-{\mathrm{Placebo}}_{{\mathrm{baseline}}}\right)/2\end{array}$$

5 $$\begin{array}{l}\Delta \Delta {\mathrm{FC}}_{{\mathrm{week24}}-{\mathrm{week52}}}=\left({\mathrm{Felzartamab}}_{{\mathrm{week52}}}-{\mathrm{Felzartamab}}_{{\mathrm{week24}}}\right)/2\\\qquad\qquad\qquad\qquad\;-\,\left({\mathrm{Placebo}}_{{\mathrm{week52}}}-{\mathrm{Placebo}}_{{\mathrm{week24}}}\right)/2\end{array}$$

6 $$\begin{array}{l}\Delta \Delta {\mathrm{FC}}_{{\mathrm{baseline}}-{\mathrm{week52}}}=\left({\mathrm{Felzartamab}}_{{\mathrm{week52}}}-{\mathrm{Felzartamab}}_{{\mathrm{baseline}}}\right)/2\\\qquad\qquad\qquad\qquad\quad-\left({\mathrm{Placebo}}_{{\mathrm{week52}}}-{\mathrm{Placebo}}_{{\mathrm{baseline}}}\right)/2.\end{array}$$

#### Functional enrichment analysis

Geneset enrichment analysis of GO, DOSE, KEGG, Reactome, MsigDB and WikiPathways terms and a de novo injury ontology based on all marker genes from AKI-induced cell states (that is, ‘New’) from healthy and injured kidney allografts^20^ was carried out on differentially expressed genes identified by equations (4)–(6) (*P* < 0.05) using ClusterProfiler v.4.12.6 (refs. ^40,41^), DOSE v.3.30.1 and ReactomePA v.1.48.0 packages in R. Simplified interpretations were manually defined using semantic similarities in their pathways descriptions as well as the core enrichment genes contributing to the enrichment of each pathway.

### Reporting summary

Further information on research design is available in the Nature Portfolio Reporting Summary linked to this article.

## Online content

Any methods, additional references, Nature Portfolio reporting summaries, source data, extended data, supplementary information, acknowledgements, peer review information; details of author contributions and competing interests; and statements of data and code availability are available at 10.1038/s41591-025-03653-3.

## Supplementary information

Supplementary Information Supplementary Tables 1–7.

Reporting Summary

## Figures and Tables

**Extended Data Table 1:**
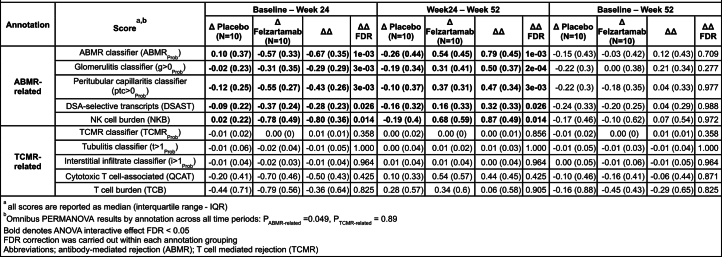
Effect of felzartamab on molecular scores

**Extended Data Table 2:**
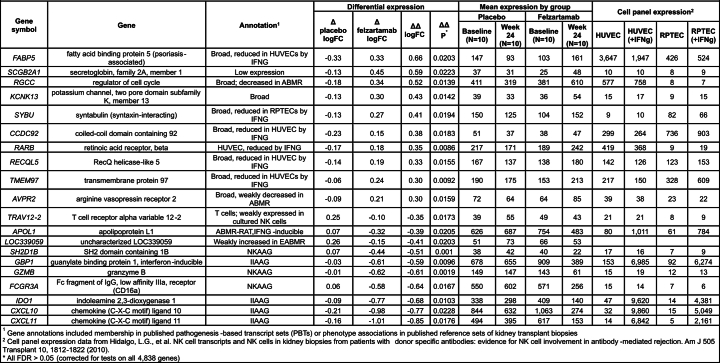
Top 20 ranked genes between baseline and week24 in biopsies from placebo and felzartamab treated patients sorted by treatment effect (selected by ΔΔ p, sorted by ΔΔlogFC)

**Extended Data Table 3:**
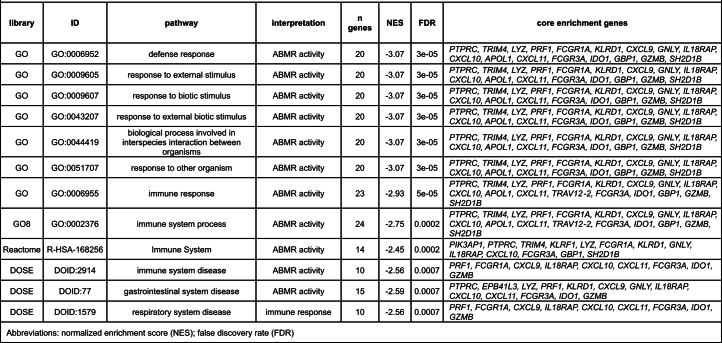
Functional enrichment analysis of genes affected by felzartamab treatment between baseline and week 24 (by FDR)

**Extended Data Table 4:**
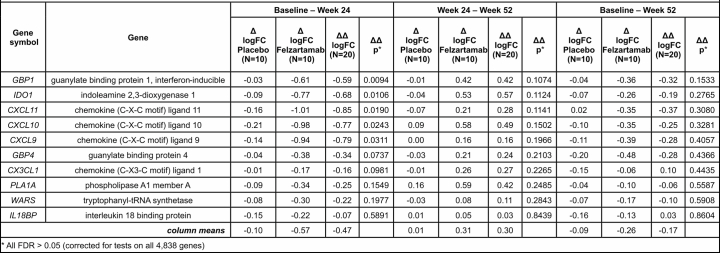
Effect of treatment on interferon gamma-inducible antibody-mediated rejection activity genes (IIAAG)

**Extended Data Table 5:**
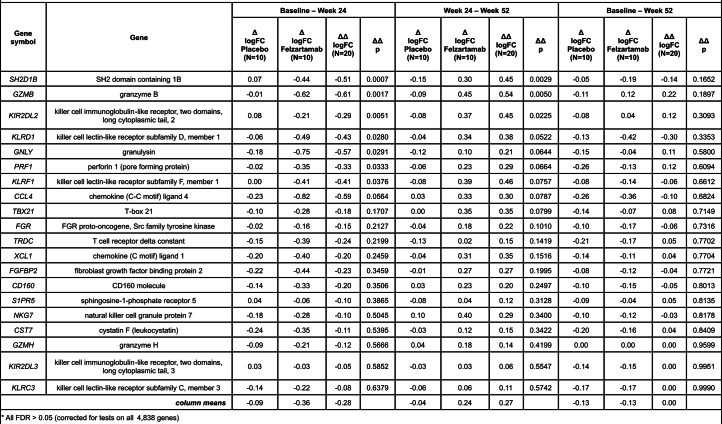
Effect of treatment on natural killer (NK) cell-expressed antibody-mediated rejection activity genes (NKAAG)

**Extended Data Table 6:**
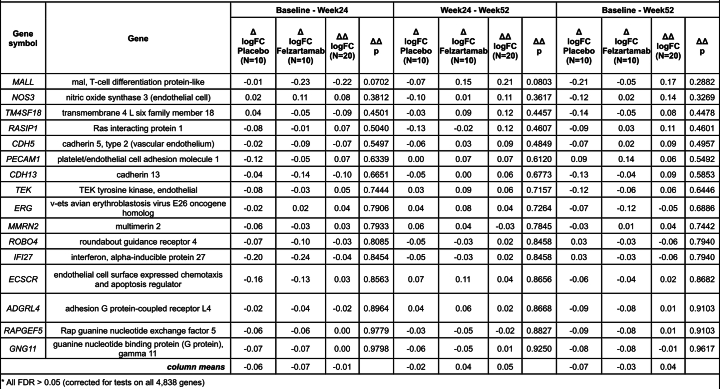
Effect of treatment on antibody-mediated rejection-associated endothelial genes (AEG)

**Extended Data Table 7:**
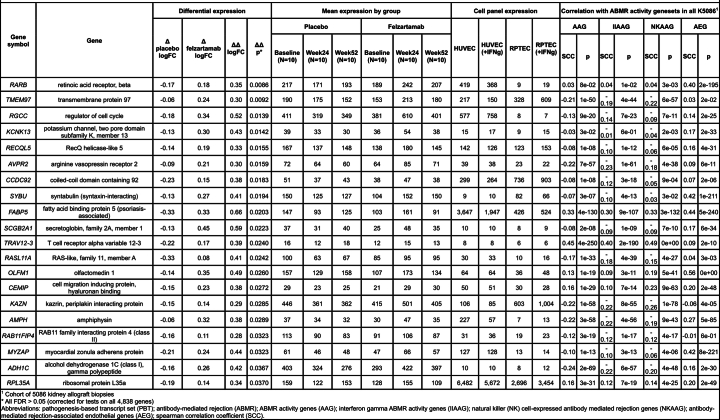
Top 20 ranked genes increased by felzartamab between baseline and week 24 (by P-value)

**Extended Data Table 8:**
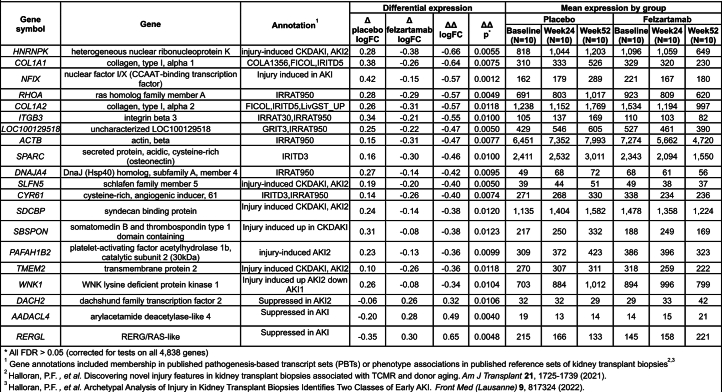
Top 20 ranked genes between baseline and week 52 in biopsies from placebo and felzartamab treated patients (selected by P-value, sorted by ΔΔlogFC)

**Extended Data Table 9:**
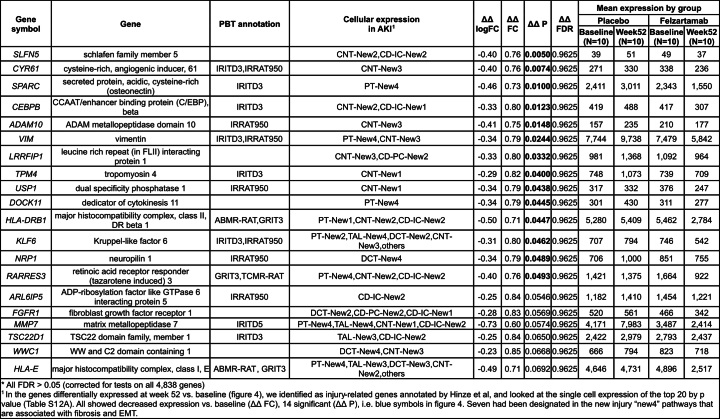
Top 20 ranked Hinze-defined injury genes between baseline and week 52 biopsies from placebo and felzartamab treated patients (by P-value)

**Extended Data Table 10:**
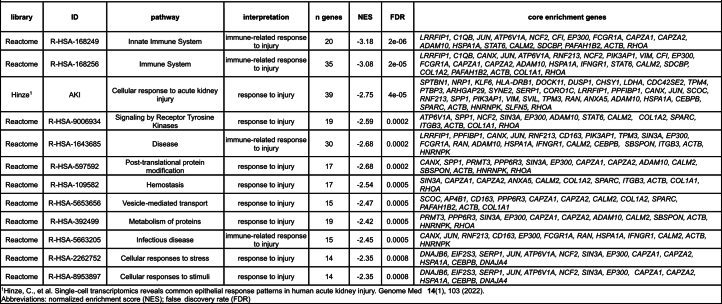
Functional enrichment analysis of genes affected by felzartamab treatment between baseline and week 52 (by FDR)

## Data Availability

The deidentified patient dataset can be obtained via Dr. Georg Böhmig (georg.boehmig@meduniwien.ac.at) 1 year after marketing authorization of felzartamab. All .CEL files from microarrays are available at Gene Expression Omnibus GSE275824. The data required to reproduce all analyses in the Article are available along with the GitHub repository associated with code used for the study via GitHub at https://github.com/TSI-PTG/CD38-effect-of-treatment/. Databases used for GO pathway analyses are publicly available: GO (https://www.geneontology.org/), DOSE (https://bioconductor.org/packages/release/bioc/html/DOSE.html), KEGG (https://www.genome.jp/kegg/), Reactome (https://curator.reactome.org/), MsigDB (https://www.gsea-msigdb.org/gsea/msigdb) and WikiPathways (https://www.wikipathways.org/).

## Code availability

All R code used for these analyses is available via GitHub at https://github.com/TSI-PTG/CD38-effect-of-treatment/. A step-by-step guide to the main analyses is available at https://tsi-ptg.github.io/CD38-effect-of-treatment/.
