# Supplementary material for: Effect of felzartamab on the molecular phenotype of antibody-mediated rejection in kidney transplant biopsies
Source: Nat Med. 2025 Apr 29;31(5):1668–76. doi: 10.1038/s41591-025-03653-3 (PMC12092283; doi:10.1038/s41591-025-03653-3)
Supplement: Supplementary file 1 — Supplementary Tables 1–7. [file 41591_2025_3653_MOESM1_ESM.pdf]

nature medicine

Supplementary information https://doi.org/10.1038/s41591-025-03653-3

Effect of felzartamab on the molecular phenotype of antibody-mediated rejection in kidney transplant biopsies

In the format provided by the authors and unedited

Table of Contents

Supplementary Table 1. Demographic and characteristics of the participants at baseline* 2
Supplementary Table 2. MMDx signouts in patients who received felzartamab 3
Supplementary Table 3. Molecular scores (PBTs, classifiers) 4
Supplementary Table 4. Definition of ABMR activity genes.. 5
Supplementary Table 5. IFNG-inducible ABMR activity genes 6
Supplementary Table 6. Definition of NK cell-expressed ABMR activity genes 7
Supplementary Table 7. ABMR-associated endothelial genes 8

Supplementary Table 1. Demographic and characteristics of the participants at baseline*			
**Variables**	**Placebo (n=10)**	**Felzartamab (n=10)**	**All patients (N=20)**
**Recorded at transplantation**			
Female sex – no. (%)	7 (70.0)	3 (30.0)	10 (50.0)
Median recipient age (IQR) – yr	50 (17-59)	34 (5-58)	41 (5-59)
Mean recipient age (SD) - yr	45 (13)	35 (15)	40 (14)
Living donor – no. (%)	2 (20.0)	3 (30.0)	5 (25.0)
Median donor age (range) – yr	36 (3-63)	48 (18-55)	43 (3-63)
Mean donor age (SD) – yr	37 (18)	42 (14)	39 (16)
Median HLA (A, B, DR) mismatch (IQR)	3 (2 to 3)	2 (2 to 4)	3 (2 to 3)
**Recorded at trial inclusion**			
Median age of study patients (range) – yr	58 (36-74)	44 (31-65)	53 (31-74)
Mean age of study patients (SD) – yr	57 (11)	45 (11)	51 (12)
Median time to inclusion in the trial (IQR) – yr	11 (6 to 19)	8 (5 to 11)	9 (5 to 19)
Median eGFR (IQR) – mL/min/1.73 m2	36 (33 to 46)	63 (34 to 70)	37 (33 to 66)
Mean eGFR (SD) – mL/min/1.73 m2	42 (19)	54 (20)	48 (20)
Median protein/creatinine ratio (IQR) – mg/g	1227 (178 to 1475)	830 (421 to 1364)	993 (196 to 1489)
Mean protein/creatinine ratio (SD) – mg/g	1101 (1081)	1414 (1686)	1257 (1387)
Banff 2019 ABMR phenotypes (baseline biopsies) – # (%)			
Active ABMR	3 (30.0)	4 (40.0)	7 (35.0)
Chronic active ABMR	7 (70.0)	6 (60.0)	13 (65.0)
Additional borderline lesion	2 (20.0)	0 (0.0)	2 (10.0)
MMDx signouts – no. (%)			
Moderate to severe early-stage ABMR	1 (10.0)	1 (10.0)	2 (10.0)
Severe early-stage ABMR	1 (10.0)	1 (10.0)	2 (10.0)
Moderate fully-developed ABMR	2 (20.0)	2 (20.0)	4 (20.0)
Severe fully-developed ABMR	4 (40.0)	4 (40.0)	8 (40.0)
Late-stage ABMR	1 (10.0)	2 (20.0)	3 (15.0)
Mixed rejection	1 (10.0)	0 (0.0)	1 (5.0)
DSA characteristics (screening visit)			
HLA class I DSA only – no. (%)	3 (30.0)	4 (40.0)	7 (35.0)
HLA class II DSA only – no. (%)	6 (60.0)	5 (50.0)	11 (55.0)
HLA class I and II DSA – no. (%)	1 (10.0)	1 (10.0)	2 (10.0)
Anti-DQ DSA – no. (%)	5 (50.0)	5 (50.0)	10 (50.0)
Peak MFI of DSA >10,000 – no. (%)	3 (30.0)	4 (40.0)	7 (35.0)
Median DSA (IQR) – n	2 (1 to 3)	2 (1 to 3)	2 (1 to 3)

*Modified from Mayer et al. 2024 N Engl J Med 391;2

Abbreviations: ABMR, antibody-mediated rejection; DSA, donor-specific antibody; eGFR, estimated glomerular filtration rate; HLA, human leukocyte antigen; IQR, interquartile range; MFI, mean fluorescence intensity; SD, standard deviation

Supplementary Table 2. MMDx signouts in patients who received felzartamab			
**Patient**	**Baseline biopsy**	**Week 24 biopsy (end of felzartamab treatment)**	**Week 52 biopsy (28 weeks after felzartamab treatment)**
2	Severe FABMR	Moderate FABMR (Rej score is normal)	Severe FABMR
4	FABMR (99% medulla)	Late ABMR (mild) (90% medulla)	Late ABMR (minimal)
5	Severe FABMR	Moderate EABMR	Moderate EABMR
7	FABMR (92% medulla)	NR	Moderate/Severe FABMR
9	LABMR (minimal)	NR (LABMR+cg score)	NR
12	FABMR (99% medulla)	NR (99% medulla)	Possible ABMR (Rej score is normal)
13	Severe EABMR	Moderate EABMR	Severe FABMR
16	Severe FABMR	Mild ABMR (Rej score is normal) (99% medulla)	Severe EABMR
19	LABMR (mild)	NR (50% medulla)	Moderate/Severe FABMR
22	EABMR	NR	Mild EABMR

Supplementary Table 3. Molecular scores (PBTs, classifiers)		
**Category**	**Abbreviation**	**Description**
**TCMR-related**	TCMR-RAT	TCMR-associated [1]
	QCAT	Cytotoxic T cell associated [2]
	TCB	T-cell burden related [3]
	$i>1_{Prob}$	Top 20 transcripts in classifier of probability of histologic kidney i-lesion score > 1 [4]
	$t>1_{Prob}$	Top 20 transcripts in classifier of probability of histologic kidney t-lesion score > 1 [4]
	$TCMR_{Prob}$	Top 20 transcripts in classifier of probability of histologic kidney TCMR diagnosis [4]
**ABMR-related**	NKB	Natural killer (NK) cell burden [5] (only valid if there is no TCMR)
	DSAST	Kidney derived DSA selective transcripts [3]
	$ABMR_{Prob}$	Top 20 transcripts in classifier of probability of histologic kidney ABMR diagnosis [4]
	$g>0_{Prob}$	Top 20 transcripts in classifier of probability of histologic g-lesion score > 0 [4]
	$ptc>0_{Prob}$	Top 20 transcripts in classifier of probability of histologic ptc-lesion score > 0 [4]
**New ABMR-related PBTs**	AAG	ABMR-associated activity genes
	IIAAG	IFNG-inducible ABMR-associated activity genes
	NKAAG	NK cell expressed ABMR-associated activity genes
	AEG	ABMR-associated endothelial cell genes
**Macrophage-related**	AMAT1	Alternatively activated macrophage transcripts [6]
	QCMAT	Constitutive macrophage transcripts [7]
**Recent injury-related**	IRITD3	Injury-repair induced, day 3 (IRITD3) [8]
	IRITD5	Injury-repair induced, day 5 (IRITD3) [8]
	IRRAT30	Injury-repair associated (IRRAT30) [9]
**Atrophy-fibrosis-related**	$ci>1_{Prob}$	Fibrosis classifier [10]
	$ct>1_{Prob}$	Atrophy classifier [10]
**Normal parenchyma**	KT1	Normal kidney transcripts (except solute carriers) [11]
	KT2	Normal kidney solute carrier transcripts [11]

[A] https://www.ualberta.ca/medicine/institutes-centres-groups/atagc/research/gene-lists.

Reference List

1. Halloran, P.F., Venner, J.M. & Famulski, K.S. Comprehensive Analysis of Transcript Changes Associated With Allograft Rejection: Combining Universal and Selective Features. *Am J Transplant* **17**, 1754-1769 (2017).
2. Hidalgo, L.G., *et al.* The transcriptome of human cytotoxic T cells: measuring the burden of CTL-associated transcripts in human kidney transplants. *Am J Transplant* **8**, 637-646 (2008).
3. Hidalgo, L.G., *et al.* NK cell transcripts and NK cells in kidney biopsies from patients with donor-specific antibodies: evidence for NK cell involvement in antibody-mediated rejection. *Am J Transplant* **10**, 1812-1822 (2010).
4. Reeve, J., *et al.* Assessing rejection-related disease in kidney transplant biopsies based on archetypal analysis of molecular phenotypes. *Jci Insight* **2**, e94197 (2017).
5. Hidalgo, L.G., *et al.* Interpreting NK cell transcripts versus T cell transcripts in renal transplant biopsies. *Am J Transplant* **12**, 1180-1191 (2012).
6. Famulski, K.S., Sis, B., Billesberger, L. & Halloran, P.F. Interferon-gamma and donor MHC class I control alternative macrophage activation and activin expression in rejecting kidney allografts: a shift in the Th1-Th2 paradigm. *Am J Transplant* **8**, 547-556 (2008).
7. Famulski, K.S., *et al.* Defining the canonical form of T-cell-mediated rejection in human kidney transplants. *Am J Transplant* **10**, 810-820 (2010).
8. Famulski, K.S., *et al.* Transcriptome analysis reveals heterogeneity in the injury response of kidney transplants. *Am J Transplant* **7**, 2483-2495 (2007).
9. Famulski, K.S., *et al.* Molecular phenotypes of acute kidney injury in kidney transplants. *J Am Soc Nephrol* **23**, 948-958 (2012).
10. Halloran, P.F., *et al.* Molecular phenotype of kidney transplant indication biopsies with inflammation in scarred areas. *Am J Transplant* **19**, 1356-1370 (2019).
11. Einecke, G., Broderick, G., Sis, B. & Halloran, P.F. Early loss of renal transcripts in kidney allografts: relationship to the development of histologic lesions and alloimmune effector mechanisms. *Am J Transplant* **7**, 1121-1130 (2007).

Abbreviations: pathogenesis-based transcript set (PBT); donor-derived cell-free DNA (dd-cfDNA); antibody-mediated rejection (ABMR); T cell mediated rejection (TCMR)

0

Supplementary Table 4. Definition of ABMR activity genes

Gene symbol	Gene	PBT annotation	Cell panel expression[1]					Correlation with ABMR in K5086[2]				Correlation with dd-cfDNA in N604[3]	
			NK	CD4	CD8	HUVEC (unstimulated)	HUVEC (IFNg stimulated)	SCC EABMR K5086	p EABMR K5086	SCC FABMR K5086	p FABMR K5086	SCC dd-cfDNA (cp/mL) N604	p dd-cfDNA (cp/mL) N604
FGFBP2	fibroblast growth factor binding protein 2	ABMR-RAT,DSAST	7,651	102	913	11	12	0.46	1e-259	0.48	2e-298	0.47	2e-21
GNLY	granulysin	ABMR-RAT,DSAST,QCAT	11,561	5,121	8,299	35	34	0.38	7e-175	0.46	5e-268	0.52	2e-26
CXCL11	chemokine (C-X-C motif) ligand 11	ABMR-RAT,GRIT3	9	7	7	14	6,842	0.31	4e-112	0.48	9e-290	0.58	5e-35
S1PR5	sphingosine-1-phosphate receptor 5	ABMR-RAT	347	19	18	31	32	0.31	4e-112	0.48	4e-299	0.50	1e-24
PLA1A	phospholipase A1 member A	ABMR-RAT,DSAST,GRIT3	18	13	15	17	545	0.29	1e-100	0.63	0e+00	0.58	2e-34
CXCL10	chemokine (C-X-C motif) ligand 10	ABMR-RAT,GRIT1	20	21	18	32	9,860	0.29	4e-98	0.45	3e-251	0.58	2e-34
CXCL9	chemokine (C-X-C motif) ligand 9	ABMR-RAT,GRIT1	27	18	23	41	11,888	0.29	5e-97	0.47	3e-275	0.58	8e-34
CCL4L1	chemokine (C-C motif) ligand 4-like 1	ABMR-RAT						0.28	6e-90	0.44	2e-239	0.54	4e-29
KLRD1	killer cell lectin-like receptor subfamily D, member 1	ABMR-RAT	1,465	22	119	26	22	0.28	2e-89	0.47	1e-283	0.55	5e-30
CCL4	chemokine (C-C motif) ligand 4	ABMR-RAT	3,200	266	1,977	35	30	0.27	2e-85	0.49	3e-308	0.60	5e-37
CCL3	chemokine (C-C motif) ligand 3	ABMR-RAT	622	243	2,098	18	15	0.26	2e-80	0.41	3e-210	0.55	7e-31
WARS	tryptophanyl-tRNA synthetase	ABMR-RAT,GRIT3	344	337	302	910	10,642	0.26	2e-78	0.49	0e+00	0.62	4e-41
NKG7	natural killer cell granule protein 7	ABMR-RAT,QCAT	8,535	369	3,138	21	25	0.26	1e-76	0.42	2e-222	0.50	5e-24
PRF1	perforin 1 (pore forming protein)	ABMR-RAT,QCAT	6,787	598	1,344	50	60	0.26	2e-76	0.49	7e-300	0.55	1e-30
GZMB	granzyme B	ABMR-RAT,QCAT	10,203	1,634	4,546	15	19	0.25	4e-74	0.45	2e-247	0.57	2e-32
GBP1	guanylate binding protein 1, interferon-inducible	ABMR-RAT,GRIT3	742	617	529	153	6,985	0.24	7e-68	0.44	1e-241	0.58	2e-34
IDO1	indoleamine 2,3-dioxygenase 1	ABMR-RAT,GRIT3	38	35	31	47	9,620	0.24	3e-66	0.48	4e-299	0.58	4e-35
GBP4	guanylate binding protein 4	ABMR-RAT,GRIT1,GRIT3	270	130	101	31	1,721	0.23	2e-63	0.49	7e-301	0.54	8e-29
RARRES3	retinoic acid receptor responder (tazarotene induced) 3	GRIT3,TCMR-RAT	3,162	1,403	1,427	44	3,157	0.23	3e-63	0.34	4e-136	0.50	2e-24
GZMH	granzyme H	RAT	7,166	90	1,351	27	24	0.23	2e-62	0.34	5e-135	0.39	2e-14
							average	0.28		0.46		0.54	

[1] Cell panel expression data from Hidalgo, L.G., et al. NK cell transcripts and NK cells in kidney biopsies from patients with donor specific antibodies: evidence for NK cell involvement in antibody-mediated rejection. Am J 505 Transplant 10, 1812-1822 (2010).
[2] Cohort of 5086 kidney allograft biopsies. Halloran, P.F., *et al.* Subthreshold rejection activity in many kidney transplants currently classified as having no rejection. Am J Transplant **25**, 72-87 (2025).
[3] Cohort of 604 kidney allograft biopsies and paired blood samples for donor-derived cell-free DNA (dd-cfDNA) testing. Gauthier et al. Distinct Molecular Processes Mediate Donor-derived Cell-free DNA Release From Kidney Transplants in Different Disease States. Transplantation **108**, 898-910 (2023).
Abbreviations: pathogenesis-based transcript set (PBT); natural killer (NK); antibody-mediated rejection (ABMR); early ABMR (EABMR); human umbilical vein endothelial cell (HUVEC); interferon gamma (IFNg); spearman correlation coefficient (SCC).

Supplementary Table 5. IFNG-inducible ABMR activity genes

Gene symbol	Gene	PBT annotation	Cell panel expression[1]					Correlation with ABMR in K5086[2]				Correlation with dd-cfDNA in N604[3]	
			NK	CD4	CD8	HUVEC (unstimulated)	HUVEC (IFNg stimulated)	SCC EABMR K5086	p EABMR K5086	SCC FABMR K5086	p FABMR K5086	SCC dd-cfDNA (cp/mL) N604	p dd-cfDNA (cp/mL) N604
CXCL9	chemokine (C-X-C motif) ligand 9	ABMR-RAT,GRIT1	27	18	23	41	11,888	0.29	5e-97	0.47	3e-275	0.58	8e-34
WARS	tryptophanyl-tRNA synthetase	ABMR-RAT,GRIT3	344	337	302	910	10,642	0.26	2e-78	0.49	0e+00	0.62	4e-41
CXCL10	chemokine (C-X-C motif) ligand 10	ABMR-RAT,GRIT1	20	21	18	32	9,860	0.29	4e-98	0.45	3e-251	0.58	2e-34
IDO1	indoleamine 2,3-dioxygenase 1	ABMR-RAT,GRIT3	38	35	31	47	9,620	0.24	3e-66	0.48	4e-299	0.58	4e-35
GBP1	guanylate binding protein 1, interferon-inducible	ABMR-RAT,GRIT3	667	752	598	127	8,616	0.24	7e-68	0.44	1e-241	0.58	2e-34
CXCL11	chemokine (C-X-C motif) ligand 11	ABMR-RAT,GRIT3	9	6	6	12	7,317	0.31	4e-112	0.48	9e-290	0.58	5e-35
IL18BP	interleukin 18 binding protein	GRIT1,TCMR-RAT	168	70	67	70	3,270	0.22	1e-55	0.41	6e-200	0.50	8e-25
GBP4	guanylate binding protein 4	ABMR-RAT,GRIT3	270	130	101	31	1,721	0.23	2e-63	0.49	7e-301	0.54	8e-29
PLA1A	phospholipase A1 member A	ABMR-RAT, DSAST,GRIT3	18	13	15	17	545	0.29	1e-100	0.63	0e+00	0.58	2e-34
CX3CL1	chemokine (C-X3-C motif) ligand 1	ABMR-RAT,IRITD3	24	27	22	22	183	0.23	9e-61	0.49	0e+00	0.44	2e-18
							average	0.26		0.48		0.56	

[1] Cell panel expression data from Hidalgo, L.G., et al. NK cell transcripts and NK cells in kidney biopsies from patients with donor specific antibodies: evidence for NK cell involvement in antibody-mediated rejection. Am J 505 Transplant 10, 1812-1822 (2010).
[2] Cohort of 5086 kidney allograft biopsies. Halloran, P.F.*, et al.* Subthreshold rejection activity in many kidney transplants currently classified as having no rejection. Am J Transplant **25**, 72-87 (2025).
[3] Cohort of 604 kidney allograft biopsies and paired blood samples for donor-derived cell-free DNA (dd-cfDNA) testing. Gauthier et al. Distinct Molecular Processes Mediate Donor-derived Cell-free DNA Release From Kidney Transplants in Different Disease States. Transplantation **108**, 898-910 (2023).
Abbreviations: pathogenesis-based transcript set (PBT); natural killer (NK); antibody-mediated rejection (ABMR); early ABMR (EABMR); human umbilical vein endothelial cell (HUVEC); interferon gamma (IFNg); spearman correlation coefficient (SCC).

2

Supplementary Table 6. Definition of NK cell-expressed ABMR activity genes

Gene symbol	Gene	PBT annotation	Cell panel expression[1]					Correlation with ABMR in K5086[2]				Correlation with dd-cfDNA in N604[3]	
			NK	CD4	CD8	HUVEC (unstimulated)	HUVEC (IFNg stimulated)	SCC EABMR K5086	p EABMR K5086	SCC FABMR K5086	p FABMR K5086	SCC dd-cfDNA (cp/mL) N604	p dd-cfDNA (cp/mL) N604
PRF1	perforin 1 (pore forming protein)	ABMR-RAT,QCAT	16,872	2,596	4,955	50	56	0.26	2e-76	0.49	7e-300	0.55	1e-30
GNLY	granulysin	ABMR-RAT,DSAST,QCAT	11,561	5,121	8,299	35	34	0.38	7e-175	0.46	5e-268	0.52	2e-26
GZMB	granzyme B	ABMR-RAT,QCAT	10,203	1,634	4,546	15	19	0.25	4e-74	0.45	2e-247	0.57	2e-32
TRDC	T cell receptor delta constant	ABMR-RAT	9,333	214	372	19	20	0.21	3e-52	0.47	2e-280	0.48	2e-22
NKG7	natural killer cell granule protein 7	ABMR-RAT,QCAT	8,535	369	3,138	21	25	0.26	1e-76	0.42	2e-222	0.50	5e-24
FGFBP2	fibroblast growth factor binding protein 2	ABMR-RAT,DSAST	7,651	102	913	11	12	0.46	1e-259	0.48	2e-298	0.47	2e-21
CST7	cystatin F (leukocystatin)	ABMR-RAT,QCAT	7,421	2,469	2,904	22	28	0.18	8e-38	0.41	1e-203	0.49	3e-23
KLRF1	killer cell lectin-like receptor subfamily F, member 1	ABMR-RAT,DSAST,NKB	7,393	21	16	8	11	0.22	2e-54	0.49	0e+00	0.44	1e-18
GZMH	granzyme H	RAT	7,166	90	1,351	27	24	0.23	2e-62	0.34	5e-135	0.39	2e-14
KLRD1	killer cell lectin-like receptor subfamily D, member 1	ABMR-RAT	6,493	49	754	22	36	0.28	2e-89	0.47	1e-283	0.55	5e-30
SH2D1B	SH2 domain containing 1B	ABMR-RAT,DSAST,NKB	5,392	10	13	17	16	0.20	5e-45	0.50	0e+00	0.38	4e-14
S1PR5	sphingosine-1-phosphate receptor 5	ABMR-RAT	3,808	22	32	29	27	0.31	4e-112	0.48	4e-299	0.50	1e-24
XCL1	chemokine (C motif) ligand 1	ABMR-RAT	3,683	242	1,491	23	32	0.22	1e-54	0.45	3e-249	0.49	2e-23
CCL4	chemokine (C-C motif) ligand 4	ABMR-RAT	3,200	266	1,977	35	30	0.27	2e-85	0.49	3e-308	0.60	5e-37
FGR	FGR proto-oncogene, Src family tyrosine kinase	cIRIT	3,104	33	48	57	46	0.16	7e-32	0.43	8e-228	0.49	1e-23
CD160	CD160 molecule	ABMR-RAT	1,833	14	23	18	21	0.20	3e-45	0.42	1e-213	0.37	1e-13
TBX21	T-box 21	ABMR-RAT	1,803	165	408	35	41	0.22	9e-58	0.47	1e-280	0.47	2e-21
KIR2DL2	killer cell immunoglobulin-like receptor, two domains, long cytoplasmic tail, 2		1,563	26	38	40	37	0.19	1e-43	0.32	5e-121	0.26	3e-07
KLRC3	killer cell lectin-like receptor subfamily C, member 3	ABMR-RAT	1,317	11	273	11	11	0.18	3e-40	0.42	8e-213	0.40	3e-15
KIR2DL3	killer cell immunoglobulin-like receptor, two domains, long cytoplasmic tail, 3		1,293	29	33	48	41	0.19	4e-41	0.25	3e-72	0.23	6e-06
							mean	0.24		0.44		0.46	

[1] Cell panel expression data from Hidalgo, L.G., et al. NK cell transcripts and NK cells in kidney biopsies from patients with donor specific antibodies: evidence for NK cell involvement in antibody-mediated rejection. Am J 505 Transplant 10, 1812-1822 (2010).
[2] Cohort of 5086 kidney allograft biopsies. Halloran, P.F., *et al.* Subthreshold rejection activity in many kidney transplants currently classified as having no rejection. Am J Transplant **25**, 72-87 (2025).
[3] Cohort of 604 kidney allograft biopsies and paired blood samples for donor-derived cell-free DNA (dd-cfDNA) testing. Gauthier et al. Distinct Molecular Processes Mediate Donor-derived Cell-free DNA Release From Kidney Transplants in Different Disease States. Transplantation **108**, 898-910 (2023).
Abbreviations: pathogenesis-based transcript set (PBT); natural killer (NK); antibody-mediated rejection (ABMR); early ABMR (EABMR); human umbilical vein endothelial cell (HUVEC); interferon gamma (IFNg); spearman correlation coefficient (SCC).

Supplementary Table 7. ABMR-associated endothelial genes

Gene symbol	Gene	PBT annotation	Cell panel expression[1]					Correlation with ABMR in K5086[2]				Correlation with dd-cfDNA in N604[3]	
			NK	CD4	CD8	HUVEC (unstimulated)	HUVEC (IFNg stimulated)	SCC EABMR K5086	p EABMR K5086	SCC FABMR K5086	p FABMR K5086	SCC dd-cfDNA (cp/mL) N604	p dd-cfDNA (cp/mL) N604
GNG11	guanine nucleotide binding protein (G protein), gamma 11	ABMR-RAT,DSAST	66	23	25	5,182	4,144	-0.01	4e-01	0.45	6e-256	0.23	5e-06
PECAM1	platelet/endothelial cell adhesion molecule 1	ABMR-RAT,ENDAT,IRRAT950	344	105	350	3,178	3,034	-0.01	6e-01	0.41	2e-202	0.41	7e-16
CDH5	cadherin 5, type 2 (vascular endothelium)	ABMR-RAT,DSAST, ENDAT	19	17	17	2,845	2,224	0.14	2e-24	0.46	3e-260	0.37	3e-13
ECSCR	endothelial cell surface expressed chemotaxis and apoptosis regulator	ABMR-RAT,cIRIT	17	40	38	2,710	2,493	0.03	2e-02	0.47	2e-272	0.37	2e-13
ROBO4	roundabout guidance receptor 4	ABMR-RAT,DSAST	27	43	41	1,694	1,486	0.20	2e-47	0.55	0e+00	0.38	7e-14
ERG	v-ets avian erythroblastosis virus E26 oncogene homolog		19	10	11	1,038	995	-0.02	2e-01	0.42	7e-217	0.22	2e-05
MALL	mal, T-cell differentiation protein-like	ABMR-RAT,DSAST	29	23	27	802	360	0.11	5e-15	0.46	2e-269	0.36	2e-12
RASIP1	Ras interacting protein 1	ABMR-RAT,ENDAT	43	26	38	635	446	0.06	3e-06	0.50	0e+00	0.16	3e-03
TEK	TEK tyrosine kinase, endothelial	ABMR-RAT,DSAST, ENDAT	12	10	11	567	367	0.07	1e-07	0.40	7e-197	0.09	7e-02
MMRN2	multimerin 2	ABMR-RAT	10	8	10	539	453	0.04	1e-02	0.43	7e-223	0.18	6e-04
TM4SF18	transmembrane 4 L six family member 18	ABMR-RAT,DSAST	15	14	16	415	389	-0.01	3e-01	0.40	5e-198	0.28	5e-08
RAPGEF5	Rap guanine nucleotide exchange factor 5	ABMR-RAT	13	17	29	301	151	0.09	1e-10	0.43	1e-229	0.30	8e-09
NOS3	nitric oxide synthase 3 (endothelial cell)	ENDAT	42	27	24	209	181	0.08	8e-09	0.42	8e-212	0.25	1e-06
CDH13	cadherin 13	ABMR-RAT,DSAST	11	9	10	127	125	-0.05	4e-04	0.41	7e-206	0.30	5e-09
							average	0.05		0.44		0.28	

[1] Cell panel expression data from Hidalgo, L.G., et al. NK cell transcripts and NK cells in kidney biopsies from patients with donor specific antibodies: evidence for NK cell involvement in antibody-mediated rejection. Am J 505 Transplant 10, 1812-1822 (2010).
[2] Cohort of 5086 kidney allograft biopsies. Halloran, P.F., *et al.* Subthreshold rejection activity in many kidney transplants currently classified as having no rejection. Am J Transplant **25**, 72-87 (2025).
[3] Cohort of 604 kidney allograft biopsies and paired blood samples for donor-derived cell-free DNA (dd-cfDNA) testing. Gauthier et al. Distinct Molecular Processes Mediate Donor-derived Cell-free DNA Release From Kidney Transplants in Different Disease States. Transplantation **108**, 898-910 (2023).
Abbreviations: pathogenesis-based transcript set (PBT); natural killer (NK); antibody-mediated rejection (ABMR); early ABMR (EABMR); human umbilical vein endothelial cell (HUVEC); interferon gamma (IFNg); spearman correlation coefficient (SCC).
