# Supplementary material for: Effect of felzartamab on the molecular phenotype of antibody-mediated rejection in kidney transplant biopsies
Source: Nat Med. 2025 Apr 29;31(5):1668–76. doi: 10.1038/s41591-025-03653-3 (PMC12092283; doi:10.1038/s41591-025-03653-3)
Supplement: Supplementary file 2 — Reporting Summary [file 41591_2025_3653_MOESM2_ESM.pdf]

nature portfolio

Corresponding author(s): Halloran PF, Bohmig GA

Last updated by author(s): February 26, 2025

Reporting Summary

Nature Portfolio wishes to improve the reproducibility of the work that we publish. This form provides structure for consistency and transparency in reporting. For further information on Nature Portfolio policies, see our Editorial Policies and the Editorial Policy Checklist.

Statistics

For all statistical analyses, confirm that the following items are present in the figure legend, table legend, main text, or Methods section.

n/a	Confirmed	
☐	☒	The exact sample size (*n*) for each experimental group/condition, given as a discrete number and unit of measurement
☐	☒	A statement on whether measurements were taken from distinct samples or whether the same sample was measured repeatedly
☐	☒	The statistical test(s) used AND whether they are one- or two-sided<br>*Only common tests should be described solely by name; describe more complex techniques in the Methods section.*
☐	☒	A description of all covariates tested
☐	☒	A description of any assumptions or corrections, such as tests of normality and adjustment for multiple comparisons
☐	☒	A full description of the statistical parameters including central tendency (e.g. means) or other basic estimates (e.g. regression coefficient) AND variation (e.g. standard deviation) or associated estimates of uncertainty (e.g. confidence intervals)
☐	☒	For null hypothesis testing, the test statistic (e.g. *F*, *t*, *r*) with confidence intervals, effect sizes, degrees of freedom and *P* value noted<br>*Give P values as exact values whenever suitable.*
☒	☐	For Bayesian analysis, information on the choice of priors and Markov chain Monte Carlo settings
☐	☒	For hierarchical and complex designs, identification of the appropriate level for tests and full reporting of outcomes
☐	☒	Estimates of effect sizes (e.g. Cohen's *d*, Pearson's *r*), indicating how they were calculated

Our web collection on statistics for biologists contains articles on many of the points above.

Software and code

Policy information about availability of computer code

Data collection	All clinical data were manually collected and tabulated. All gene expression data were collected using Affymetrix GeneChip Command Console Scan Control version4 .0.0.1567
Data analysis	All data analysis and figure production was carried out in R statistical software version4 .3.3, using publicly available libraries including the BioBase v2.64.0, vegan v2.6.8, limma v3.60.4, lmerTest v3.l.3, ggeffects vl.7.1, ClusterProfiler v4.12.6, DOSE v3.30.l, ReactomePA v 1.48.0, ARTool v0.11.1, and performance v0.12.3 libraries. All R code used for these analyses is accessible in a public GitHub repository. https://github.com/TSI-PTG/CD38-effect-of-treatment/

For manuscripts utilizing custom algorithms or software that are central to the research but not yet described in published literature, software must be made available to editors and reviewers. We strongly encourage code deposition in a community repository (e.g. GitHub). See the Nature Portfolio guidelines for submitting code & software for further information.

Data

Policy information about availability of data

All manuscripts must include a data availability statement. This statement should provide the following information, where applicable:

- Accession codes, unique identifiers, or web links for publicly available datasets
- A description of any restrictions on data availability
- For clinical datasets or third party data, please ensure that the statement adheres to our policy

The de-identified patient data set can be obtained via Dr. Georg B6hmig (georg.boehmig@meduniwien.ac.at) one year after marketing authorization of felzartamab. Databases used for gene ontology pathway analyses are publicly available; GO (https://www.geneontology.org/), DOSE (https://bioconductor.org/packages/release/bioc/html/DOSE.html), KEGG (https://www.genome.jp/kegg/), Reactome (https://curator.reactome.org/), MsigDB (https://www.gsea-msigdb.org/gsea/msigdb), and Wiki Pathways (https://www.wikipathways.org/).

Research involving human participants, their data, or biological material

Policy information about studies with human participants or human data. See also policy information about sex, gender (identity/presentation), and sexual orientation and race, ethnicity and racism.

Reporting on sex and gender	Patient sex was collected and reported in the study but no sex-based analyses were carried out in the study, in part because of insufficient sample sizes.
Reporting on race, ethnicity, or other socially relevant groupings	Patient self-reporting on race was included but not used for any analyses in the study.
Population characteristics	Relevant covariates in the study which were not part of the recruitment criteria included patient age, sex, ethnicity, time to inclusion in the trial post-transplant, donor age, whether the donor was deceased or alive during allocation, and genotypic mismatch of the donor/recipient (human leukocyte antigen matching), but no analyses were carried out on these covariates, in part because of insufficient sample sizes.
Recruitment	Eligible patients were identified upon routine monitoring of transplant recipients in outpatient care. Standardized protocols for surveillance and indication allograft biopsies ensured timely diagnosis of ABMR and thus adequate patient enrollment to reach the target sample size. There were no self-selection biases involved in recruitment.
Ethics oversight	Ethic approval was obtained from the institutional ethics committees of the Medical University of Vienna and Charite University Medicine Berlin, respectively. Written, informed consent to participate were obtained from all participants by appointed investigators, who had been trained beforehand according to GCP standards. The study was conducted in accordance with the principles of the Declaration of Helsinki 2008. The authors adhered to all the trial-related requirements, Good Clinical Practice (GCP) requirements (ICH GCP), Good Laboratory Practice (GLP) and the applicable regulatory requirements. The trial is registered with EUDRACT (EudraCT number: 2021-000545-40) and ClinicalTrials.gov (NCT05021484).

Note that full information on the approval of the study protocol must also be provided in the manuscript.

Field-specific reporting

Please select the one below that is the best fit for your research. If you are not sure, read the appropriate sections before making your selection.

☒ Life sciences ☐ Behavioural & social sciences ☐ Ecological, evolutionary & environmental sciences

For a reference copy of the document with all sections, see nature.com/documents/nr-reporting-summary-flat.pdf

Life sciences study design

All studies must disclose on these points even when the disclosure is negative.

Sample size	The trial included 22 subjects (during the final recruitment phase (December 2022), an amendment expanded the number of participants from 20 to 22 to accommodate eligible patients undergoing concurrent screening). Details of the clinical trail protocol are also described in: Mayer et al. Trials. 2022 Apr 8;23(1):270. doi: 10.1186/sl3063-022-06198-9; Mayer et al. N Engl J Med. 2024 Jul 11;391(2):122-132 (including Suppl Appendix and Statistical Analysis Plan).
Data exclusions	Two patients were excluded from the study due to incomplete sampling. We have reported and discussed this in the manuscript.
Replication	Replication included 11 patients in either placebo or felzartamab arms of the study. Replication was successful for 10 patients in each plecebo and treatment arms (see Data exclusions for details). A single biopsy per patient per follow-up date was assessed by microarray. Technical reproducibility in microarray assays is high (~99%).
Randomization	1:1 randomization was performed using a computerized assignment through a web-based platform (www.meduniwien.ac.at/randomizer)

Randomization | (felzartamab versus placebo). Permuted block randomization with block size 2 was employed, taking into account stratification by study site (Vienna versus Berlin) and AMR categories (active versus chronic active AMR).

Blinding | Roles for investigators, study nurses, and clinical pharmacists were defined within the online randomization tool. To maintain blinding, patients, care providers, and those assessing outcomes remained unaware of the randomization sequence. An independent, non-blinded study pharmacist generated the allocation sequence and prepared study medication and placebo. The investigational drug and placebo was formulated to be identical in color, appearance and smell. Study physicians and nurses were provided with blinded medication for administration. Throughout the trial, participating investigators, medical staff, and patients remained blinded to treatment arm allocation until the completion of the last patient's participation.

Reporting for specific materials, systems and methods

We require information from authors about some types of materials, experimental systems and methods used in many studies. Here, indicate whether each material, system or method listed is relevant to your study. If you are not sure if a list item applies to your research, read the appropriate section before selecting a response.

Materials & experimental systems

n/a	Involved in the study	
☐	☒	Antibodies
☒	☐	Eukaryotic cell lines
☒	☐	Palaeontology and archaeology
☒	☐	Animals and other organisms
☐	☒	Clinical data
☒	☐	Dual use research of concern
☒	☐	Plants

Methods

n/a	Involved in the study	
☒	☐	ChIP-seq
☒	☐	Flow cytometry
☒	☐	MRI-based neuroimaging

Antibodies

Antibodies used | Felzartamab (Hi-Bio, Inc./Biogen) is a recombinant fully human monoclonal CD38 antibody (IgGI) derived from a proprietary antibody phage library, initially developed for the treatment of multiple myeloma, and being evaluated in myeloma, autoimmune diseases and transplantation (Raab et al. Lancet Haematol. 2020 May;7(5):e381-e394; Mayer et al. N Engl J Med. 2024 Jul 11;391(2):122-132; ClinicalTrials.gov: NCT06064929; NCT05065970; NCT04893096; NCT06285201; NCT04145440).

Validation | Raab et al. Lancet Haematol. 2020 May;7(5):e381-e394; Mayer et al. N Engl J Med. 2024 Jul 11;391(2):122-132; ClinicalTrials.gov: NCT06064929;NCT05065970;NCT04893096;NCT06285201;NCT04145440)

Clinical data

Policy information about clinical studies
All manuscripts should comply with the ICMJE guidelines for publication of clinical research and a completed CONSORT checklist must be included with all submissions.

Clinical trial registration | The trial is registered with EUDRACT (EudraCT number: 2021-000545-40) and ClinicalTrials.gov (NCT05021484)

Study protocol | The study protocol is published: Mayer et al. Trials. 2022 Apr 8;23(1):270. doi: 10.1186/s13063-022-06198-9; Mayer et al. N Engl J Med. 2024 Jul 11;391(2):122-132 (including Suppl Appendix and Statistical Analysis Plan)

Data collection | The data for the investigator-initiated, phase 2 pilot felzartamab trial were collected at the Medical University of Vienna, Austria and Charite Universitatsmedizin Berlin, Germany. Patient recruitment occurred between October 2021 and March 2023, with data collection extending until March 2024. The study involved kidney transplant recipients who met the following inclusion criteria: voluntary written informed consent, age >18 years (maximum 80 years), functioning living or deceased donor allograft ?180 days post-transplantation, an eGFR ?20 ml/min/1.73 m2 (CKD-EPI formula), presence of HLA class I and/or II antigen-specific antibodies (preformed and/or de novo DSA), active or chronic/active ABMR (±C4d in PTC) according to the Banff 2019 classification, and a molecular ABMR score (MMDx) ?0.2. Participants were followed for 52 weeks. Further informations are provided in Mayers et alTrials. 2022 Apr 8;23(1):270. doi: 10.1186/s13063-022-06198-9; Mayer et al. N Engl J Med. 2024 Jul 11;391(2):122-132 (including Suppl Appendix and Statistical Analysis Plan)

Outcomes | The primary outcome was the safety and tolerability of felzartamab in kidney transplant recipients with ABMR on baseline immunosuppression. Secondary outcomes included assessments of DSA and immunoglobulin levels (at weeks 0, 12, 24, and 52), immunodominant DSA (mean fluorescence intensity, dilution-based changes, and number detected), and total Ig/IgG subclasses. Additional outcomes involved evaluating leukocyte subsets, circulating immune cells, and CD38 expression using flow cytometry, as well as results from protocol biopsies (at weeks 24 and 52), focusing on ABMR classification, inflammation scores (g+ptc, cg, interstitial fibrosis), and molecular analyses (MMDx and rejection-related classifiers). Biomarkers such as CXCL9, CXCLl0, BAFF, and TTV were measured alongside clinical parameters, including eGFR slope, iBox scores, proteinuria, and 12-month graft/patient survival. Further informations are provided in Mayers et al Trials. 2022 Apr 8;23(1):270. doi: 10.1186/ s13063-022-06198-9; Mayer et al. N Engl J Med. 2024 Jul 11;391(2):122-132 (including Suppl Appendix and Statistical Analysis Plan)

Plants

Seed stocks	*Report on the source of all seed stocks or other plant material used. If applicable, state the seed stock centre and catalogue number. If plant specimens were collected from the field, describe the collection location, date and sampling procedures.*
Novel plant genotypes	*Describe the methods by which all novel plant genotypes were produced. This includes those generated by transgenic approaches, gene editing, chemical/radiation-based mutagenesis and hybridization. For transgenic lines, describe the transformation method, the number of independent lines analyzed and the generation upon which experiments were performed. For gene-edited lines, describe the editor used, the endogenous sequence targeted for editing, the targeting guide RNA sequence (if applicable) and how the editor was applied.*
Authentication	*Describe any authentication procedures for each seed stock used or novel genotype generated. Describe any experiments used to assess the effect of a mutation and, where applicable, how potential secondary effects (e.g. second site T-DNA insertions, mosiacism, off-target gene editing) were examined.*
